# Combined group and individual therapy for patients with avoidant personality disorder—A pilot study

**DOI:** 10.3389/fpsyt.2023.1181686

**Published:** 2023-05-04

**Authors:** Theresa Wilberg, Geir Pedersen, Kjetil Bremer, Merete Selsbakk Johansen, Elfrida Hartveit Kvarstein

**Affiliations:** ^1^Division of Mental Health and Addiction, Department of Research and Innovation, Oslo University Hospital, Oslo, Norway; ^2^Institute of Clinical Medicine, University of Oslo, Oslo, Norway; ^3^Section for Personality Psychiatry and Specialized Treatments, Division of Mental Health and Addiction, Oslo University Hospital, Oslo, Norway; ^4^The Norwegian Centre of Mental Disorders Research (NORMENT), Institute of Clinical Medicine, University of Oslo, Oslo, Norway; ^5^South-Eastern Norway Regional Health Authority, Unit for Mental Health Care and Substance Abuse Treatment, Hamar, Norway

**Keywords:** avoidant personality disorder, group therapy, combined therapy, outcome, follow-up

## Abstract

**Objective:**

Avoidant personality disorder (AvPD) is a common disorder within mental health services, associated with significant psychosocial impairment. The disorder has been neglected in research. There are currently no evidence-based treatments for AvPD, and there is a need for treatment studies focusing particularly on this form of personality pathology. The present study was a pilot study of combined group and individual therapy for patients with AvPD, based on mentalization-based and metacognitive interpersonal therapy. The aim was to investigate the feasibility of the treatment program and the course of symptoms and personality functioning during treatment and 1-year follow-up.

**Methods:**

The study included 28 patients. Clinical evaluation at baseline comprised structured diagnostic interviews and patients' self-report of symptoms, psychosocial function, interpersonal problems, personality functioning, alexithymia, self-esteem, attachment style, therapeutic alliance, and client satisfaction. Patients' self-report were repeated at the end of treatment and 1-year follow-up.

**Results:**

The drop-out rate was 14%. Average treatment length among the 22 treatment completers was 17 months. Mean levels of therapeutic alliance and client satisfaction were satisfactory. Effect sizes were large for global symptom distress, depression, anxiety, and psychosocial adjustment, and in the moderate range for aspects of personality functioning. Yet, the results showed a wide range of outcomes among the patients.

**Conclusions:**

This pilot study shows promising results for combined group- and individual therapy for AvPD patients with moderate to severe impairment. Larger scale studies should be conducted to increase empirically based knowledge to guide development of differentiated treatments adapted to patients' various levels of AvPD severity and profiles of personality dysfunction.

## 1. Introduction

Avoidant personality disorder (AvPD) is increasingly recognized as a disorder with significant impairment in many patients and a challenge regarding development of helpful treatments (1, 2). The disorder is characterized by a combination of poor self-esteem and a pervasive pattern of social withdrawal, and is among the most common personality disorders (PDs). The mean reported population prevalence in western countries is 3.7% (3, 4). In studies of clinical outpatient samples within specialist mental health services, reported frequencies range from 44 to 55% (5–7). Patients usually present with a range of co-occurring conditions like depression, anxiety, eating and substance use disorders, and have increased risk of physical health problems (2, 8, 9). It is thus an important task for mental health services to develop efficient treatment offers.

By definition, a diagnosis of AvPD implies a pervasive pattern of social inhibition, feeling of inadequacy, and hypersensitivity to negative evaluation (10). The diagnostic criteria include avoidance of activities and social interaction, as well as restraint in intimate relationships. Yet such features encompass various levels of severity of personality functioning, ranging from mild to severe impairments. Recent research indicate that many patients struggle with their sense of identity and self-agency, impaired emotional awareness including weak experiences of positive affects like enjoyment, playfulness and curiosity, and impaired ability to monitor their own mental states (2, 11–15). Poor reflective functioning both regarding own and others mind are characteristic of patients with severe disorder (16–18). Further, fearful and detached attachment, emotional distancing, lack of feeling close or connected, difficulties sharing personal material and deficient social learning experiences are part of the core problems of many patients (19–23). Such features may vary among patients, but will influence the patients' motivation for treatment, their ability to form a working alliance with a therapist or therapy group, and the therapeutic processes (24–26).

There is, however still a scarcity of treatment research focusing on AvPD (27). Only four randomized controlled trials (RCT) with a small number of patients have focused particularly on AvPD (28–31), and some additional natural design studies (32–35). A few RCTs have been conducted on cluster C personality disorders which have included more than 60% patients with AvPD (36, 37). Various treatment approaches like graded exposure, social skills training, cognitive behavior therapy, schema therapy, psychodynamic therapy, supportive expressive therapy, mentalization-based therapy and combined metacognitive and mentalization-based therapy have been reported helpful, but there are few studies. A striking overall finding is the heterogeneity of outcomes, ranging from remission after short-term treatment to poor response in even longer-term treatments. Such differences may reflect that some treatment approaches are more efficient than other treatments. Yet as for psychotherapy more generally, patient characteristics probably account for a significant amount of outcome variance (38). Thus, a single theoretical approach and treatment duration is not compatible for all patients. It is especially those with moderate or severe personality dysfunction that represent a challenge to mental health services, many of whom will need longer-term treatments. Due to a lack of research we are far from empirically based knowledge of “what works for whom” regarding patients with AvPD. In contrast to borderline personality disorder (BPD), for which there are several available evidence based treatments (39), service providers and therapists have little empirical evidence to lean on regarding developing adapted treatment for patients with AvPD.

Interestingly, quite a few of AvPD treatment studies concern group therapy, or group therapy as an add-on to individual therapy (28, 30, 33, 35, 40, 41). Besides the potential cost-effectiveness of the group format more generally, group therapy could be particularly helpful for patients with AvPD because of their relational difficulties, and patients are often referred to groups due to a stalemate in individual therapy. The group setting represents an arena for interpersonal exposure with potentials for corrective experiences of their deflated self-image and rigid representations of others as critical. If experienced as safe enough, groups may facilitate emotional sharing, interpersonal trust, a sense of connection, and social learning, which give opportunities for new perspectives on self and others and reduced interpersonal avoidance. On the other hand, as group therapy may be too anxiety provoking for some patients with poor affect regulation and impaired reflective functioning (16), it is suggested that combined individual therapy could help patients make use of their group therapy. A recent RCT of schema therapy over 2 years for patients with BPD found that the combination of group and individual therapy was superior to a predominantly group format both regarding treatment retention and reduction of BPD pathology (42). So far, only one study has investigated a systematic combination of group an individual therapy for patients with AvPD. Simonsen et al. (32) included 30 patients to mentalization-based (MBT) group therapy combined with individual metacognitive interpersonal therapy (MIT), with promising results for those who completed treatment. More studies are needed to explore the feasibility and potentials of combined group and individual therapy for patients with AvPD.

Reduction of symptom distress and improved psychosocial function are valuable goals in treatment of patients with AvPD. More ambitious goals of improving the underlying personality problems may be more difficult to achieve, particularly for those with severe disorder, but are important as it may protect against recurrence of symptom disorders and reduced life quality in the longer run. According to the dimensional approach to personality disorder in the DSM-5 Alternative Model of Personality Disorders (AMPD) (43) and ICD-11 (44) personality pathology is conceptualized as different degrees of impairments in level of personality functioning in the self and relational domains. Among the central aspects of personality functioning of relevance to AvPD are self-esteem, affect awareness, ability to self-reflect, and the capacity for intimate or enduring relationships. Thus, treatment studies should investigate to what degree patients change toward more adaptive personality functioning in such areas.

The present study was a pilot project set up to gain experience with treating AvPD patients in a program focusing particularly on AvPD. The background was a general scarcity of treatment research on AvPD (27), and the project was as an initial step toward developing an offer of specially adapted treatment within the specialist mental health system. The treatment was a time-limited combined group- and individual therapy program. The aims of the present study were to explore (a) the feasibility of the treatment in terms of completion, drop-out, attendance, client satisfaction and therapeutic alliance, and (b) the course of symptoms, psychosocial adjustment and various aspects of personality functioning during treatment and 1-year follow-up.

## 2. Methods

### 2.1. Setting and design

The pilot study was conducted during the period from 2012 to 2019. The study took place at the Outpatient Clinic for Specialized Treatment of Personality Disorders, Oslo University Hospital, which is a treatment setting specialized in MBT for patients with BPD (45, 46). The pilot AvPD program was a small part of the activity at the outpatient clinic, only one group of maximum eight–nine patients was treated at a time.

The pilot project had a prospective naturalistic longitudinal design including a 1-year follow-up evaluation. Patients had given informed written consent to participate in the study. The project was approved by the Regional Committee for Medical Research Ethics, South East Norway.

### 2.2. Treatment

#### 2.2.1. Treatment modality

The treatment modality was combined weekly group- and individual therapy. Based on existing treatment research, which were all studies of short-term treatments, we initially chose a time-limited one-year program with a closed group format. However, clinical impression and preliminary data from the first two groups of patients indicated that the treatment length was too short. Consequently, the treatment was extended to a 2-year program, and after 6 months of treatment, patients in the second group were offered to continue for a maximum of 2 years treatment. By this, the treatment changed to a slow open program, i.e., when patients ended their treatment new patients were admitted to the group. The treatment was performed by a small team, which comprised the group and individual therapists. The team met once a week for administrative purposes and video-based supervision.

#### 2.2.2. Theoretical orientation

The psychotherapy was inspired by MBT (45, 47) and MIT (48). MBT is a specialized form of psychodynamic therapy originally developed for patients with BPD. The therapists took part in weekly supervision of MBT with patients with BPD in the clinic, as well as yearly supervision by Anthony Bateman who was pivotal in developing MBT for BPD. The target in MBT is the patient's mentalizing difficulties, believed to be rooted in early attachment relationships. For patients with BPD their mentalizing ability is typically lost when experiencing strong affects in close relationships. The therapy focuses on situations in the patient's life when mentalizing is limited or failing, applying interventions like empathic validation, collaborative exploration of interpersonal episodes, and interventions to expand mentalizing (45). MBT instructs therapists to be reticent using interpretations and instead adopt a not-knowing attitude. Regarding AvPD, poorly functioning patients may have more profound and persistent mentalizing difficulties linked to a general low affect consciousness and problems accessing their own mental states (15, 18). MBT is not yet manualized to adapt to AvPD, but the team applied central MBT principles such as a focus on the patient's mental state, challenging patients to self-reflect when exploring concrete episodes in the patient's current life, adopting a therapist not-knowing stance, and keeping an affect and relational focus.

MIT is an eclectic treatment focusing on human interpersonal motivational systems, e.g., attachment, social rank, autonomy, and how experiences with caregivers and peers become internalized as interpersonal schemas (48). Interpersonal schemas refer to representations of self and others, and expected responses from others. Such representations influence the person's behaviors, which in turn work back on the person's self-image. Patients with AvPD have several maladaptive schemas resulting in negative interpersonal patterns, which contribute to maintenance or worsening of their problems. Most patients, however, also have more healthy schemas, albeit not easy to access, that might be strengthened and expanded during therapy. The MIT guidelines are suitable for personality disordered patients with problems with overregulation and inhibition, like AvPD (49). MIT and MBT have several features in common which make it possible to combine these approaches. The concepts mentalizing in MBT and metacognition in MIT both refer to the patients' reflection on their own and others mind. Moreover, both MIT and MBT focus on mental states, affects, relationships and exploration of concrete episodes in the patient's life. In the present study MIT was not applied systematically, but the team found the MIT model's focus on motivational systems, maladaptive interpersonal schemas and the emphasis on positive affects as helpful additional perspectives treating AvPD patients. From 2015 the team received digital supervision from Giancarlo Dimaggio, one of the key contributors to the development of MIT, four–eight times a year based on case presentations.

#### 2.2.3. Group therapy

The therapy group comprised five–nine patients and was run by two co-therapists. The weekly 1 ½ h group session was structured in line with MBT groups for BPD (45, 47). It started with a short summary from the group last week, then a go-around, asking if someone had something to bring forward to the group, e.g., an event, situation, or a particular problem, followed by efforts to explore the event or situation. Diagnostically homogeneous groups of patients with moderate to severe AvPD are characterized by high levels of anxiety. Typical is patients' extensive use of avoidance strategies, which inhibits open interaction and sharing of personal material (50). The group therapists needed to be active to prevent long periods of silence, with continuous effort to help the patients bring forth personal experiences and further, not only relate to the therapists but also interact with each other. To counteract their habitual avoidance the patients were explicitly expected, as part of their treatment plan to bring in a personal episode or event at least every third session. The group was seen as an arena for interpersonal exposure. In addition, the patients were encouraged to expose themselves in interpersonal situations in their ordinary life between treatment sessions.

#### 2.2.4. Individual therapy

The individual therapist was not one of the group therapists. A main purpose of the weekly 45 min individual sessions was to support the patients' participation in the group therapy, in order to help them attend and use the group for their own benefit despite feeling insecure.

An important part of the individual therapy was therefore to discuss the patient's group experiences. In collaboration, the patient and individual therapist could plan themes or interpersonal episodes the patient could bring forward in the group. Afterwards they could explore how the patient experienced this sharing or the experience of not daring to share. The clinical experience, however, was that the individual therapy was an important part of the program in its own right, with the opportunity for the patient to gradually share personal topics or more nuances in their histories and current problems which they were not able to discuss in the group.

#### 2.2.5. Psychoeducation

Both the 1-year and the 2-year programs contained elements of psychoeducation. In the 1-year program treating patients in a closed group, the group therapy started with 5 weeks of weekly 1-h psychoeducation. The group then changed to a 1 ½ h weekly dynamic group in line with the description above. As the group therapy was transformed into a slow open group the rest of the sample was offered 3–4 weekly adjunctive psychoeducation group sessions of 1–1 ½ h duration in parallel with the combined individual group therapies. Subjects of the psychoeducation were attachment, affects, mentalizing, interpersonal schemas, AvPD, avoidance strategies, anxiety, depression, and psychotherapy.

#### 2.2.6. Therapists

The treatment team was multidisciplinary and always counted 4–5 group- and individual therapists. Ten therapists joined the team for a shorter or longer period during the pilot treatment 2012–2018; two psychiatric nurses, three psychiatrists, three clinical psychologists and two specializing in clinical psychology.

### 2.3. Procedures

Information of the pilot project was published on the clinic's website, and also sent to outpatient psychiatric clinics in the region. The patients were referred from outpatient psychiatric clinics, private practitioners with contracts with the regional health authorities, or general practitioners. On referral, the patients filled in a self-application containing information of their family background, education and work history, social network, former treatment and current problems. Before entering treatment, they went through 4–6 h of diagnostic and clinical evaluation based on clinician–ratings and patients' self-report of symptoms, including self-harm, suicidal ideation and behavior, psychosocial function, interpersonal problems, personality functioning, alexithymia, self-esteem and attachment stile. Patients' self-report were repeated at the end of treatment and a 1-year follow-up evaluation. As a main rule, the clinician who would become the patient's individual therapist was the one who evaluated the patient. The patient and individual therapists agreed on two–four individualized treatment foci for the patient to work on in the individual and group therapy.

#### 2.3.1. One-year follow-up

The 1-year follow-up evaluation was conducted 1 year after the treatment was planned to end. This means that patients in the 1-year program were interviewed 2 years after treatment start whereas patients in the 2-year program were interviewed 3 years after treatment start. The follow-up comprised the former self-report questionnaires and in addition, the use of health services during the follow-up period. The follow-up also included a qualitative interview focusing on the patients subjective experiences of change and their reflections on what might have contributed to such changes or lack of changes, reported in a separate study (manuscript in preparation).

### 2.4. Assessments

#### 2.4.1. Diagnoses

Diagnoses were assessed according to DSM-IV (51). Symptom disorders were based on the Mini International Neuropsychiatric Interview (M.I.N.I.) for Axis I diagnoses (52). PDs were assessed based on the SCID-II interview (53) for Axis II diagnoses. The diagnostic evaluation was made by experienced clinicians taking part in the AvPD team. The clinicians had received systematic training in diagnostic interviews and principles of the Longitudinal, Expert, All-Data (LEAD) procedure (54, 55). This means that diagnoses were based on all available information including referral letters, self-reported history and complaints, and overall clinical impression, in addition to the diagnostic interviews. The reliability of the diagnoses was not tested. In a former study using SCID-II and M.I.N.I. in this treatment unit, acceptable diagnostic reliability was indicated (56, 57).

#### 2.4.2. Psychosocial adjustment

Psychosocial adjustment was assessed by the Work and Social Adjustment Scale (WSAS) (58), a self-report 5-item scale of functional impairment that measures the level of impairment on a scale from 0 to 8, with 0 indicating no impairment at all and 8 indicating very severe impairment. The scores on the five different items are summarized in a total score of 0–40. The WSAS constitutes a reliable instrument, measuring the individual variation in a clinically important aspect of impairment. Scale reliability (Cronbach's alpha) (59) of the Norwegian version in a sample from the Norwegian Network for Personality Disorders was 0.79 at admission and 0.90 at discharge (60). Total scores above 30 denote severe disability, scores between 15 and 30 denote moderate impairment, and scores below 15 can be regarded as mild impairment or disability (60, 61). The patients also reported the number of months in at least 50% work or study activity during the past 12 months.

#### 2.4.3. Symptom distress

Global symptom distress was assessed by the Symptom Checklist-90-Revised comprising 90 items where the intensity of symptoms is rated on a 0–4 scale (score 0: “not at all,” score 4: “extremely”) and summarized in the Global Severity Index (GSI) (SCL-90-R) (62). Scale reliability (59) of GSI in a clinical population in the Norwegian Network for Personality Disorders was 0.97 (63). The cutoff for non-clinical value is 0.80 based on a Norwegian sample (64). In addition we report results on the Brief Symptom Inventory (BSI-18) (65) which is a shortened form of the SCL-90-R comprising 18 item BSI-18 and includes an overall severity index, the mean sum-score (BSI) and three subscales, i.e., depression, anxiety, and somatization.

#### 2.4.4. Social anxiety

The Liebowitz Social Anxiety Scale self-report scale (LSAS-SR) (66) consists of 24 items depicting different social situations. For each situation the patient is asked to rate their level of fear/anxiety (LSAS-fear) on a four point scale ranging from 0 (none) to 3 (severe), and avoidance (LSAS-avoidance) on a four-point scale ranging from 0 (never) to 3 (usually). The scale has demonstrated good psychometric properties in patients with social phobia with scale reliability (59) ranging from 0.91 to 0.95 for the fear, avoidance, and total score respectively (67).

#### 2.4.5. Self-esteem

The Rosenberg Self-Esteem Scale (RSES) (68) is a widely used self-report measure of global self-esteem. It contains 10 general statements assessing the degree to which respondents are satisfied with and feel good about themselves. Items are scored on a five-point Likert-scale, ranging from 1 (strongly disagree) to 5 (strongly agree) with negative statements reversed. Higher scores represent higher self-esteem. Scale reliability (59) of the Norwegian version among psychiatric outpatients was 0.90 (69).

#### 2.4.6. Alexithymia

The 20 item Toronto Alexithymia Scale (TAS-20) (70) is a self-report questionnaire rated on a five-point response format ranging from 1 to 5. In addition to the three domains (difficulty identifying feelings, difficulty describing feelings, externally oriented thinking), a total sum score is computed based on all 20 items ranging from 20 to 100. Scale reliability of the total score as estimated by McDonald's Omega (ωt) (71, 72) was 0.86 in a sample from the Norwegian Network of Personality Disorders (73). There are three commonly referenced thresholds for the TAS-20 total score, where the first ranges from 20 to 50 (No alexithymia), the second from 51 to 60 (Low alexithymia), and the last ranging from 61 to 100 (High alexithymia) (73).

#### 2.4.7. Interpersonal problems

To assess interpersonal problems, we used the Circumplex of Interpersonal Problems (CIP), a 0-to-4 Likert scale self-report questionnaire (74). CIP is a 48-item version of Alden et al.'s (75) Inventory of Interpersonal Problems (IIP-C). It comprises eight subscales, i.e., *domineering, vindictive, cold, socially avoidant, non-assertive, exploitive, overly nurturant, intrusive*, and an index of *mistrust*, summarized as a CIP sum-score with a scale reliability of 0.91 (59) in a Norwegian clinical sample (74). CIP sum-score correlates 0.99 with the sum-score obtained from Alden's IIP-C (74). A higher score indicate more severe interpersonal problems. Expected mean scores on IIP in a Norwegian non-clinical population are estimated to 0.53 (SD = 0.31) (74), and we thus applied a clinical/non-clinical cut-score of 0.85 for CIP.

#### 2.4.8. Personality difficulties

Severity Indices of Personality Problems (SIPP-118) is a self-report questionnaire that measures 16 facets of (mal)adaptive personality capacities (76). It is based on a dimensional approach to personality pathology and is consistent with the concept of level of personality functioning in criterion A in the DSM-5 Alternative Model of Personality Disorders (AMPD) (77). The SIPP asks respondents to rate the extent to which they agree on 118 different statements while thinking back on the past 3 months. Responses are scored on a 4-point Likert-scale, ranging from 1 (completely disagree) to 4 (completely agree). High scores on the SIPP indicate higher levels of adaptive capacities, whereas lower scores indicate more maladaptive personality functioning. The Norwegian version of the SIPP has shown good reliability at the facet level (78). In the present study, we included eight facets within the self- and interpersonal domains with particular relevance to AvPD, i.e., self-respect, stability of self-image, enjoyment, self-reflection, enduring relationships, intimacy, feelings recognized, and cooperation.

#### 2.4.9. Attachment insecurity

The self-report questionnaire Experiences in Close Relationships (ECR) (79) was applied to assess attachment stile in adult close relationships. It refers to how a patient generally experiences and feels in their current close relations and comprises two higher-order dimensions of insecure/secure attachment, i.e., attachment avoidance and attachment anxiety (80, 81). It consists of 36 statements scored on a 7-point Likert scale ranging from 1 (not true) to 7 (very much true). The measures are derived by computing the mean of the 18 items for the two subscales, with a possible range from 1 to 7. Higher scores means higher levels of attachment insecurity. Cronbach's alpha was 0.92 for the avoidance and anxiety scales in a Norwegian sample of patients with mainly PDs (82).

#### 2.4.10. Autism

We used the Autism-Spectrum Quotient (AQ) to screen for autism spectrum disorder (83). AQ comprises 50 items rated on a 4-point scale of which the total scores are computed to range from 0 to 50. A score of 32+ is considered a useful cutoff for distinguishing individuals who have clinically significant levels of autistic traits.

#### 2.4.11. Therapeutic alliance

The Working Alliance Inventory-Short Revised (WAI-SR) (84) based on the Working Alliance Inventory (85), is a 12 items self-report which includes three subscales—Goals, Tasks, and Bond—with four items for each. Each item is rated on a 7-point scale, ranging from 1 (never) to 7 (always). Higher scores indicate a better therapeutic alliance, but according to the labels on the response-format, a mean score on the scales at or above 4 signify satisfactory to excellent alliance (“Fairly Often true”). WAI-SR has demonstrated good reliability in outpatients and inpatients with α for the subscales ranging from 0.81 to 0.93 (86). Patient reported working alliance was assessed with reference to the individual therapy, as well as in relation to the group therapists.

#### 2.4.12. Client satisfaction

Client satisfaction questionnaire (CSQ-8) is designed to measure client satisfaction with services. It includes 8 items rated on a 4-point scale to produce a range of 8–32, with higher scores indicating greater satisfaction (87). Scores from 24 represents good to excellent satisfaction.

### 2.5. Participants

The inclusion criteria in the pilot study were a diagnosis of AvPD, and motivation for change and treatment focusing on interpersonal exposure inside and outside the treatment setting. To ensure that the patients had some arena for social exposure it was required that the patients had a minimum of social contact outside the family, or were in some kind of work- or study context or had realistic plans for such activities. Initially patients with age between 20 and 45 years could be included, which was later limited to age between 20 and 30 years and applied to the majority of the patients.

Exclusion criteria were co-occurring schizotypal, schizoid, paranoid, or antisocial personality disorder (PD), current alcohol or substance dependence, psychotic disorders, bipolar I disorder, severe PTSD, untreated ADHD (adult form), pervasive developmental disorder (e.g., Asperger's syndrome), organic syndromes, or any other disorder that entails total withdrawal and isolation, being homeless, and insufficient skills in Norwegian language. The patients were included during the period 2012–2016 and the 1-year follow-up investigation ended in 2019.

Twenty-eight patients were included in the pilot treatment, 18 females and 10 males, average age 27.5 years (SD = 4.7). Nine patients were offered a one-year program and 19 patients a 2-year program. One patient had subthreshold AvPD fulfilling three AvPD criteria, diagnosed as PD not otherwise specified. The patients had only one PD diagnosis, except one patient with co-occurring BPD. Despite low co-occurrence of other PD traits the sample was characterized by high levels of interpersonal problems and symptom distress, and moderate to severe social dysfunction. Suicidal ideation was prevalent, and some patients had histories of suicide attempts and self-harm. The sample was further characterized by high levels of alexithymia and significant maladaptive personality functioning both in the self and relational domains. The levels of attachment insecurity were considerable compared to Norwegian population norms (81), only four patients lived in a romantic relationship and many had been in several former treatments (see Tables 1, 3).

**Table 1:** Socio-demographic and clinical characteristics, full sample *N* = 28.

	***N* (%)**
Age, mean (SD)	27.5 (4.7)
**Sex**	
Female	18 (64)
Male	10 (36)
**Marital status**	
Single	24 (86)
Married/partnered	4 (14)
Education, no. years after junior high school, mean (SD)	5.0 (2.8)
**Work situation**	
Student or employment	19 (68)
Rehabilitation money or unemployed	9 (32)
Age at first contact with health services due to mental problems	19.4 (5.0)
No. of previous outpatient treatments, mean (SD) *n* = 27	3.1 (2.1)
Previous inpatient treatment, *n* = 27	4 (15)
Any previous suicide attempt	5 (18)
Suicide thoughts last 12 months (*n* = 26)	23 (89)
Self-harm last 12 months (*n* = 26)	5 (19)
No. PD criteria, mean (SD)	9.5 (3.9)
No. of AvPD criteria, mean (SD)	5.1 (1.1)
No. of symptom disorders, mean (SD)	1.9 (1.3)
Anxiety disorders	18 (64)
Social phobia	15 (54)
Mood disorders	14 (50)
Eating disorders	6 (21)
Attachment avoidance, mean (SD) *n* = 25	4.1 (1.2)
Attachment anxiety, mean (SD) *n* = 25	4.3 (1.3)
Autism questionnaire (AQ), mean (SD) *n* = 24	21.8 (4.9)

### 2.6. Statistics

Effect size was assessed by Cohen's d with pooled SDs correcting for uneven sample sizes. The magnitude of the effect sizes may be interpreted using Cohen's convention as small (0.2), medium (0.5), and large (0.8) (88). We used the Reliably Change Index (RCI) to calculate the number of patients who were reliably changed on GSI and CIP during treatment and follow-up (89). The RCI is an index of individual change based on the difference between two time-points divided by the standard error of this difference and taking the reliability of the measure into account. For the calculation of RCI for GSI we used the scale reliability, Cronbach's alpha (59) *r* = 0.97 based on a clinical population in the Norwegian Network for Personality Disorders (63). A change of 0.25 represented reliable change. Patients with decrease in GSI equal or above 0.25 were regarded as reliable improved, whereas those with an increase in GSI equal or above 0.25 were regarded as reliable deteriorated. The RCI for CIP was based on the scale reliability of 0.91 in a Norwegian clinical sample (74). A change of 0.41 represented reliable change. Change in WAI during treatment was tested by two-sided paired *t*-tests.

## 3. Results

### 3.1. Feasibility

Among the 28 participants two patients were asked to leave treatment. One was diagnosed with Asperger syndrome during the first year and treatment was therefore discontinued. Treatment was discontinued for one more patient due to poor attendance and severe alexithymia. One patient attempted suicide after a few months in treatment and dropped out shortly after. Altogether four patients (14%) dropped out, three after 1, 6, and 8 months. The fourth patient was formally discharged after 27 months, but was considered a dropout by the therapists, due to minimal attendance for several months. Thus 22 patients (79%) ended treatment in accordance with a joint plan between patient and therapist. The clinical course will be reported for these patients considered as treatment completers. Among them is one patient who developed paranoid symptoms on the border of psychosis, and was taken out of the group therapy and instead offered two weekly sessions of individual therapy.

Mean length of treatment for completers was 17 months (SD = 6; median 15, range 10–26). Fifteen patients (68%) had regular attendance as rated by the individual therapist, whereas seven patients (32%) had some irregularities. Average level of client satisfaction was 26 (SD = 5). The therapeutic alliance in the individual and group therapy after 3 months and at the end of treatment was on average satisfactory, but with some variance (Table 2). In two-sided paired *t*-tests, the alliance in individual therapy increased significantly for all subscales; Goal (*p* = 0.042), Task (*p* = 0.011) and Bond (*p* = 0.033), and alliance to the group therapists increased significantly for Goal (*p* = 0.042).

**Table 2:** Working alliance with individual therapist and the group therapists (WAI-SR).

	**3 months (*N* = 19) Mean (SD)**	**EOT^a^ (*N* = 20/21) Mean (SD)**
**Goal**		
Individual	5.3 (1.1)	5.7 (1.1)
Group	4.1 (1.3)	4.8 (1.5)
**Task**		
Individual	4.7 (1.2)	5.4 (1.4)
Group	4.5 (1.3)	4.7 (1.5)
**Bond**		
Individual	4.7 (1.2)	5.2 (1.0)
Group	4.7 (1.1)	4.9 (0.9)

a End of treatment.

### 3.2. Treatment during follow-up

Seventeen patients (77% of completers) attended the follow-up evaluation. Six patients (35%) had started treatment with a psychiatrist or psychologist during the follow-up period, mainly individual therapy by private practitioners. Only one patient attended group psychotherapy after end of the study treatment. One patient had two psychiatric inpatient admissions. Four patients (24%) were in treatment at the time of the follow-up investigation.

### 3.3. Course of symptoms and psychosocial adjustment

The effect sizes for general symptom distress were large for both GSI and BSI, and increased from end of treatment to follow-up for both measures (see Table 3). Effect sizes for the BSI subscales were also in the large range, except somatization, which was initially the least troublesome area of distress. Sixty-two percent of the patients were reliably improved on GSI during the treatment whereas 10% were reliably deteriorated. At follow-up 88% were reliably improved and 6% reliably deteriorated on GSI. Thirty-eight percent of the patients scored below the clinical cutoff of 0.80 for GSI at the end of treatment, and at follow-up 53% of the patients scored below this clinical cutoff. As to social anxiety, the effect size was medium for the LSAS total score during treatment, but large at follow-up, and somewhat larger for avoidance than fear.

**Table 3:** Course of symptoms and psychosocial adjustment, treatment completers (*N* = 22).

	**Baseline *N* = 22**	**EOT^a^ *N* = 21**	**Follow-up^b^ *N* = 17**	**ES^c^ Baseline—EOT (95% CI)**	**ES Baseline—follow-up (95% CI)**
	**Mean (SD)**	**Mean (SD)**	**Mean (SD)**		
Global severity index, GSI^d^	1.54 (0.51)	1.05 (0.51)	0.86 (0.56)	0.96 (0.31 to 1.57)	1.28 (0.56–1.94)
BSI total^e^	1.99 (0.76)	1.30 (0.72)	1.03 (0.83)	0.93 (0.29 to 1.54)	1.21 (0.50–1.87)
BSI depression	2.62 (0.88)	1.64 (1.01)	1.35 (1.28)	1.04 (0.38 to 1.65)	1.19 (0.48–1.84)
BSI anxiety	2.08 (0.91)	1.38 (0.79)	1.00 (0.80)	0.82 (0.18 to 1.43)	1.25 (0.54–1.91)
BSI somatization	1.26 (0.72)	0.87 (0.69)	0,74 (0.68)	0.55 (−0.07 to 1.15)	0.74 (0.07–1.38)
LSAS total^f^	82.2 (19.5) *n* = 20	71.7 (20.9) *n* = 19	58.3 (24.9)	0.52 (−0.13 to 1.15)	1.08 (0.37–1.75)
LSAS fear	42.4 (10.5) *n* = 20	38.8 (10.2) *n* = 19	32.6 (12.6)	0.31 (−0.33 to 0.94)	0.82 (0.13–1.48)
LSAS avoidance	39.8 (10.6) *n* = 20	32.9 (12.3) *n* = 19	25.7 (13.0)	0.61 (−0.05 to 1.23)	1.20 (0.48–1.88)
WSAS^g^	26.14 (6.19)	19.57 (8.58)	14.29 (10.62)	0.88 (0.24 to 1.49)	1.41 (0.68–2.09)
Patients in ≥6 month of ≥50% work/studies last year	68%	71%	76%		

a End of treatment.

b Follow-up indicates follow up one year after the one year and two year programs respectively.

c Cohens' *d* with pooled SDs and corrected for number of patients at different evaluation points.

d Global Severity Index of Symptom Checklist-90-Revised (SCL-90-R).

e Brief Symptom Inventory (BSI-18).

f Liebowitz Social Anxiety Scale - Self-Report version (LSAS-SR).

g Work and Social Adjustment Scale (WSAS).

Regarding psychosocial adjustment, the effect size for WSAS was large at the end of treatment and increased to follow-up. At the end of treatment 29% of treatment completers had scores below 15 regarded as the threshold for mild impairment or disability. At follow-up 59% scored below this 15-point criterion value. There was however only a slight increase in work and study activity. Seventy-six percent of the patients had at least 6 months of 50%−100% activity in work or studies during the follow-up period.

### 3.4. Course of personality related variables

The effect sizes on personality related variables were mainly in the medium range at the end of treatment, whereas mostly in the large range at follow-up (see Table 4). For TAS-20 the effect size was large both during treatment and at follow-up. The rate of patients with high alexithymia (TAS-20 ≥61) changed from 55% at baseline to 37 and 19% during treatment and follow-up respectively. It is noteworthy that the effect sizes were smaller regarding the interpersonal domain (CIP and SIPP) than in the self-domain (RSES and SIPP). As to interpersonal problems, 40% were reliably improved on CIP during treatment and 5% reliably deteriorated. At follow-up 47% were reliably improved and 6% reliably deteriorated on this measure. Applying a clinical cutoff of 0.85, only 15% of the patients scored below this cutoff at the end of treatment and 29% at follow-up.

**Table 4:** Course of personality related variables (*N* = 22).

	**Baseline *N* = 22**	**EOT^a^ *N =* 21**	**Follow-up^b^ *N =* 17**	**ES^c^ Baseline—EOT (95% CI)**	**ES Baseline—follow-up (95% CI)**
	**Mean (SD)**	**Mean (SD)**	**Mean (SD)**		
Self-esteem, RSES^d^	1.85 (0.55)	2.26 (0.70)	2.51 (0.84)	0.65 (0.03 to 1.25)	0.96 (0.27 to 1.60)
Alexithymia, TAS-20 total sum^e^	62.70 (8.87) *n* = 20	53.32 (13.12) *n* = 19	47.56 (13.93) *n* = 16	0.84 (0.17 to 1.48)	1.33 (0.58 to 2.02)
High alexithymia	55% *n* = 20	37% *n* = 19	19%		
Interpersonal problems, CIP^f^	1.75 (0.49)	1.53 (0.54) *n* = 20	1.23 (0.64)	0.43 (−0.19 to 1.03)	0.93 (0.25 to 1.57)
**Severity of personality problems, SIPP-118** ^g^					
**SIPP self**					
Self-respect	2.01 (0.58)	2.43 (0.72) *n* = 20	2.63 (0.86)	0.65 (0.01 to 1.25)	0.87 (0.19 to 1.51)
Stability of self-image	2.38 (0.65)	2.82 (0.56) *n* = 20	3.07 (0.58)	0.72 (0.08 to 1.33)	1.11 (0.41 to 1.77)
Enjoyment	2.20 (0.61)	2.65 (0.72) *n* = 20	2.87 (0.83)	0.68 (0.04 to 1.29)	0.94 (0.25 to 1.58)
Self-reflection	2.41 (0.50)	2.78 (0.61) *n* = 20	3.05 (0.63)	0.67 (0.03 to 1.28)	1.14 (0.44 to 1.80)
**SIPP interpersonal**					
Enduring relationships	2.30 (0.64)	2.56 (0.75) *n* = 20	2.77 (0.74)	0.37 (−0.24 to 0.98)	0.69 (0.02 to 1.32)
Intimacy	2.13 (0.63)	2.51 (0.78) *n* = 20	2.87 (0.85)	0.54 (−0.02 to 1.14)	1.01 (0.32 to 1.66)
Feeling recognized	2.62 (0.46)	2.94 (0.58) *n* = 20	3.12 (0.52)	0.61 (−0.02 to 1.22)	1.03 (0.33 to 1.68)
Cooperation	3.04 (0.45)	3.15 (0.39) *n* = 20	3.29 (0.42)	0.26 (−0.35 to 0.86)	0.57 (−0.08 to 1.21)

a End of treatment.

b Follow-up indicates follow up one year after the one year and two year programs.

c Cohens' *d* with pooled SDs and corrected for number of patients at different evaluation points.

d Rosenberg Self-Esteem Scale.

e Toronto Alexithymia Scale.

f Circumplex of Interpersonal Problems.

e Severity Indices of Personality Problems.

### 3.5. Patients offered 1 year vs. 2 year of treatment

Among treatment completers, eight patients (36%) had been offered 1 year of treatment and 14 patients (64%) had been offered a treatment length of maximum 2 years. Average treatment length was 12 months (SD = 1) and 20 months (SD = 6), respectively for those in the 1- and 2-year programs. The effect sizes during treatment were somewhat larger for those offered 2 year treatment on most central variables, except LSAS: WSAS, 0.99 vs. 0.67; GSI, 1.36 vs. 0.46; LSAS, 0.52 vs. 0.51; RSES, 0.94 vs. 0.18; TAS-20, 1.06 vs. 0.60; CIP, 0.58 vs. 0.21.

### 3.6. Investigating possible attrition bias at follow-up

To consider potential bias due to missing follow-up data, we calculated separate effect sizes for changes during treatment for the 17 patients who met for follow-up evaluation and the five patients who did not on central outcome variables. The effect sizes for those who participated in the follow-up were generally larger than for those not participating, i.e., WSAS, 0.94 vs. 0.55; GSI, 1.12 vs. 0.38; LSAS, 0.59 vs. 0.04, RSES, 0.75 vs. 0.24; TAS-20, 0.92 vs. 0.56, CIP, 0.56 vs. 0.09. The number of months in treatment did not differ between the groups, 17.1 (SD = 6.0) vs. 17.6 (SD = 7.4). Thus, those who participated in the follow-up evaluation tended to have a more favorable course during treatment.

## 4. Discussion

This was a pilot study of combined group and individual therapy for patients with AvPD. The main findings were acceptable feasibility of the program, a low drop-out rate and large to moderate effect sizes for changes in symptom distress and domains of personality functioning. The results are discussed in more detail below.

The combined group- and individual treatment was well-received by most patients. A drop-out rate of 14% is in the lower range of drop-out rates reported for PDs (26, 32, 34, 37, 90), and the majority of patients attended the treatment regularly. The two patients for whom treatment was ended by the therapists illustrates that despite a thorough initial evaluation it may sometimes be difficult to evaluate the patients' ability or motivation to make use of the treatment on beforehand. First, it may be difficult to distinguish Asperger syndrome from AvPD, as both disorders are characterized by problems with social interaction. Very inhibited AvPD patients may appear constrained in relational contexts, both regarding facial mimicry and verbal fluency, and with little eye contact. It may thus require a longer observation time to capture if such relational problems are due to autistic traits or other underlying neuropsychiatric problems. The patient in the present study who was diagnosed with Asperger syndrome during treatment had an initial AQ score well below the recommended cut-off for further assessment (83). To avoid subjecting patients to additional strain, clinicians should be attentive to this important differential diagnosis to be able to help the patients to more appropriate care as early as possible. Further, for the severely alexithymic patient with poor attendance, the present treatment approach did not match the patient's concretistic approach to own problems. The treatment was discontinued as it was not possible to engage the patient in a mental focus on social problems. The case may thus illustrate discrepancies between clinicians' wishes on behalf of the patients, and the patients' own motivation for the therapy project.

The effect sizes were large for global symptom distress, anxiety, depression and psychosocial adjustment, with further improvements during 1-year follow-up. The results for social anxiety assessed by LSAS were more moderate but was maintained or improved during follow-up. The effect sizes at follow-up may, however be overestimated due to positive selection bias at follow-up. Nevertheless, 38% of the patients were under clinical cut-off regarding GSI at the end of treatment and 29% had only mild psychosocial impairments according to WSAS, with higher rates at follow-up. Large effect sizes for symptom distress are found in other studies of AvPD as well (32, 34). Also in the cluster C-studies of Svartberg and coworkers with 62% AvPD (36) and Bamelis et al. (37) with 64% AvPD large effects sizes were found for symptom distress, both during treatment and at 2 or 3 years follow-up respectively, even if these studies did not report results specifically for AvPD. Comparisons between studies are difficult due to differences in samples, methods, and treatments. Yet, the aforementioned studies comprised treatments of 1–2 years duration, in contrast to the early RCTs of short-term treatments from the 1980s and 1990s limited to 10–14 sessions (28–30).

Despite positive changes in personality functioning, these were more moderate than for overall symptom distress, depression and anxiety, except for alexithymia. In a previous study, we found that levels of alexithymia explained much of the variance in several aspects of personality dysfunction in patients with AvPD (91). The initial high rate of alexithymia in the present study may indicate a specific severity of the present sample, and increased affect awareness and communication may therefore be an important outcome (13, 32). At follow-up most effect sizes on variables of personality functioning were in the large range, but again, these are probably somewhat overestimated due to selection bias. A few treatment studies have found significant reduction in personality pathology or have reported recovery rates from AvPD diagnosis ranging from 50 to 91% in samples of AvPD (31, 34, 35) or cluster C with more than 60% AvPD but not reporting specifically for AvPD (36, 37). The patients in the current pilot study were not re-diagnosed after treatment. However, the average levels of the SIPP facets were below the Norwegian community norms both at the termination and follow-up, indicating that even with improvement many patients still had significant personality problems (78).

Most studies of comparable treatment lengths and intensities have investigated individual therapies of different orientations. The only study of combined group and individual therapy is the pilot study of combined group MBT and individual MIT by Simonsen et al. (32), and the present results add to the empirical support of this approach. The study of Simonsen et al. however found somewhat more favorable results on measures of personality functioning, particularly regarding relational difficulties (i.e., SIPP enduring relationships and CIP interpersonal problems), which raise several questions. First, the present pilot study was the first to apply a combined MBT and MIT approach in a treatment program for patients with AvPD. The therapy was, however not manualized. Adapting MBT principles to patients with AvPD was new at the time, and experiences were gained throughout the study. Likewise, the MIT manual was published a few years into the study, the procedures were not incorporated systematically, and the team had no formal training in this approach (48). The Simonsen et al. study, initiated at a later point of time, applied the MIT principles more systematically in the individual therapy, with more supervision. As shown in Bamelis et al.' (37) study of schema therapy for PDs, more extensive therapist training may be associated with better patient outcome. Thus, more expertise, training and supervision in the specific approaches in this pilot study may probably improve outcomes.

Second, the group therapy was offered as an arena for interpersonal exposure, interaction and reflection, to help patients increase their mentalizing abilities and gain new experiences that could correct their rigid interpersonal schemas and thus increase their relational involvement in their lives. Even if the MBT principles were focused on the anxious avoidant personality pathology, the weaker outcomes on the relational domain indicate that to utilize the potentials of the group format, homogeneous AvPD groups may require more structural elements for the patients to overcome their inhibition and take the risk of more open sharing and interaction. Several techniques have been suggested for inhibited patients to reduce their inhibition, such as role play or other experiential techniques, behavioral experiments, or physical play or exercise (41, 92–94). Some techniques may be applied in group settings also in combination with psychoeducational topics, and help structure the groups and stimulate patient to interact more freely. A qualitative interview study of participants in the present pilot study indicates that for patients who benefitted from the combined therapy, the group therapy was experienced as very valuable (manuscript in preparation). Different measures to increase interpersonal engagement and group cohesion in treatment of AvPD patients should therefore be subjected to further research.

During the pilot study the group therapy was changed from a closed to a slow open group format. As patients with AvPD typically feel anxious in new relational settings, may have a general fear of novelty and need time to open up on personal matters (2), one may speculate that they prefer to attend a closed group. Some of the patients who were offered 2 year of treatment may have benefited from starting out as a closed group before gradually being exposed to new group members. We don't know to what degree this may have contributed to the better average outcomes among those who were offered 2 year of treatment. As closed groups are not feasible for long-term therapy, it is a clinical dilemma that many patients need longer-term treatment. In an RCT comparing 2 year and 6 months psychodynamic group psychotherapy, Lorentzen et al. (95) demonstrated better effectiveness for patients with PDs in the long-term treatment, both regarding general symptom distress, interpersonal problems and psychosocial functioning. Moreover, of high relevance to avoidant personality pathology was the greater improvement in self-attack, self-blame and self-neglect in the long-term groups (96).

A notable feature of the present results is the wide range of patient outcomes, as reflected in large variance in outcome variables. Some patients had very good outcome and continued to improve after therapy. The treatment may have helped them toward better dynamics, both relationally and regarding education and work. Other patients on the other hand, experienced little or no improvement, and some even deteriorated. It has been estimated that 5%−10% of patients will have higher levels of symptoms following psychotherapy more generally, and PD is one of the risk factors for detoriation or non-response (97, 98). As for BPD a recent review of 48 studies of empirically validated treatments found that on average nearly half of the patients did not respond to treatment (99). Negative experiences of psychotherapy is believed to be the result of many interacting factors such as aspects of the therapy, service structure and available treatment options, therapist competences, patient expectations and needs, and the dynamics between patient and therapist (100). This study was too small and not designed to explore such aspects.

However, even if the average therapeutic alliance in individual and group therapy was acceptable, some patients reported alliance below an acceptable level. Therapeutic alliance is a widely recognized predictor of therapy outcome, and episodes of alliance ruptures that are discussed between patient and therapist and subsequently repaired, may increase outcome (101, 102). Alliance ruptures may be of a confrontational or withdrawal nature. There is a lack of research on alliance in treatment of AvPD. But in a sample of 30 subjects with AvPD or OCPD in cognitive therapy, higher pretreatment personality pathology was associated with lower early alliance, and both higher early alliance and rupture-repair episodes predicted improvement in personality symptoms and depression (26). Establishing and maintaining a good working relationship with patients with AvPD can be challenging for patients with more severe avoidant personality pathology. In a study of individual schema therapy with patients with AvPD, Peled and colleagues found that the patients were in an avoidant detached self-state much of the time during sessions (24). Thus, engaging the patient in the therapeutic endeavor may be difficult, withdrawal ruptures could be frequent, yet difficult to detect.

We will highlight some clinical implications from this pilot study. First, the results are promising for a combined MBT and MIT oriented group- and individual therapy approach to patients with AvPD. Measures should, however be taken to develop the program further in order to improve outcome, particularly the structure of the group therapies to help group members interact and explore they interpersonal relations in the group as suggested above. Sufficient time must be spent on the initial treatment contract, and the alliance should be a continuous focus and part of the dialogue between patient and therapists. Many patients with AvPD have difficulties expressing their opinions and critics, which mean that much of the responsibility for initiating such dialogues, rely on the therapists. Like for other patients with severe PDs, therapist training and regular supervision should be part of the organizational treatment structure. Next, the range of outcomes also indicate a need for more differentiated treatment, adapted to individual patients' needs, motivations and abilities. The AvPD diagnosis encompasses a heterogeneous patient population. Some patients will have more access to their affects, more relational resources and curiosity to self-explore, and will be easier to engage in therapies based on MBT and MIT (15, 33, 103). Many patients in the present study had several previous treatments and approximately one third were in new treatments at some point during the follow-up period. Non-response to treatment may lead to patients' feeling of failure and loss of hope (100). A significant portion of patients with AvPD may need longer-term treatment than offered in this study to be able to relate to others more securely. Differentiated treatment, both regarding theoretical approach and duration, which represent real options for the therapist and patient to discuss and choose between, could reduce the risk of iatrogenic effects. A crucial problem at this point is the lack of empirically based knowledge of clinical features with relevance for treatment response. To bring the field further, more research is needed on patients with AvPD, including investigations of non-response and indices of severity of pathology.

The present study had several limitations. As a pilot study with a low a number patients, the results cannot be generalized to a broader population of patients with AvPD. Due to the naturalistic design with no control group, we cannot conclude that changes represent treatment effects. Nor can the results be attributed specifically to MBT or MIT. As the treatment was not manualized we don't know whether patient outcome was mainly related to common psychotherapeutic factors rather than the specific MBT and MIT approaches (104). Moreover, patients were offered either 1 or 2 year of treatment, but the sample was too small to investigate associations between treatment duration and outcomes in any convincing way. The better outcomes in the 2-year program may be due to other conditions than treatment duration. The inclusion of a follow-up investigation was one of the strengths of the study, however the follow-up period varied between patients in the 1 and 2 year program. The results at follow-up must also be interpreted in light of a positive selection bias. Among other strengths is the inclusion of patients with moderate to severe impairments, many of whom with several previous treatments. As a pilot study, it was successful in gaining experience with treating AvPD in a separate program, which is rare in clinical practice. It included a wide range of outcome measures, and indicated areas for further treatment development and research.

In conclusion, this small-scale pilot study indicates promising results for combined group and individual therapy for AvPD patients with moderate to severe impairment. Further development of the program may potentially improve outcomes for more patients. There is an urgent need for larger scale studies of AvPD in order to increase the empirical knowledge base to guide future development of treatments adapted to patients' various levels of severity and profiles of personality dysfunction.

## Data availability statement

The dataset presented in this article is not readily available due to restrictions imposed by the Regional Medical Ethics Committee regarding patient confidentiality. Requests to access the datasets should be directed to the hospital's Privacy and Data Protection Officer at: personvern@ous-hf.no.

## Ethics statement

The studies involving human participants were reviewed and approved by Regional Committee for Medical Research Ethics, South East Norway ref 2012/579. The patients/participants provided their written informed consent to participate in this study.

## Author contributions

TW: head of the pilot project, main responsibility for study design, data collection, data analyses, and manuscript. GP: participated in study design, main responsibility for dataprocessing, and gave substantial feedback on the manuscript. KB: participated in data collection and gave substantial feedback on the manuscript. MJ: participated in study design, data collection, and gave substantial feedback on the manuscript. EK: participated in study design, recruited patients, and gave substantial feedback on the manuscript. All authors contributed to the article and approved the submitted version.
